# An Assessment of the Etiologies Associated With Acute Abdomen Subjected to Exploratory Laparotomy: A Study From a Rural Area of Himachal Pradesh

**DOI:** 10.7759/cureus.33285

**Published:** 2023-01-02

**Authors:** Satish Kumar, Paran Tanwar, Sourabh Trivedi, Rajan Sood, Pradeep Sharma, Mukul Sharma

**Affiliations:** 1 Department of General Surgery, Dr. Rajendra Prasad Government Medical College & Hospital, Tanda, IND; 2 Department of General Surgery, Maharishi Markandeshwar Medical College & Hospital, Solan, IND; 3 Department of General Surgery, Indira Gandhi Medical College & Hospital, Shimla, IND

**Keywords:** acute abdomen, secondary care hospital, intestinal obstruction, gastrointestinal perforation, laparotomy

## Abstract

Background

The aim of this retrospective study is to establish a correlation between clinical features, surgical diagnosis, and the final diagnosis of laparotomies, as well as to establish the relationship between preoperative delay on the outcomes of surgery in the form of mortality and morbidity. Emergency surgery is high-risk in patients with acute abdomen with uncertain diagnosis. The results of surgery are remarkable and provide quick relief to the suffering and agony of patients with the dreadful condition of acute generalized peritonitis.

Methodology

Patients presenting with complaints of acute abdomen who needed laparotomy based on clinical judgment and investigations were included in this study. The study data were reviewed from April 2007 to January 2011 and March 2014 to February 2016 in a government hospital.

Results

A total of 174 patients with acute abdomen in whom there was an indication of laparotomy based on clinical judgment and radiological investigations were selected. Most patients had gastrointestinal perforation (n = 115) and acute intestinal obstruction (n = 23). The most important clinical features analyzed were abdominal tenderness (n = 160), guarding (n = 153), distention (n = 75), and tachycardia (n = 63).

Conclusions

Among the total patients, 150 underwent surgery within 24 hours of the presentation in the emergency and the remaining after 24 hours. The most common cause of laparotomy was a duodenal perforation in 79 patients and gastric perforation in 24 patients. A total of 114 patients developed no complications postoperatively. Among patients who developed postoperative complications, wound sepsis and acute respiratory distress syndrome were the most common. Mortality was noted in three patients.

## Introduction

Emergency laparotomy is the most commonly performed surgery in an emergency situation [1]. The results of surgery are remarkable and provide quick relief to the suffering and agony of patients presenting with the dreadful condition of acute generalized peritonitis. The decision to proceed with laparotomy is crucial based on surgeons’ clinical exposure and acumen. Necessary investigations also play a decisive role in laparotomy. This study focuses on the etiologies of acute abdomen leading to laparotomy at a district hospital. The findings that were noted commonly on laparotomy were hollow viscus perforation, intestinal obstruction due to various causes, and perforated appendix, among others [2,3]. Early diagnosis and timely surgical intervention are crucial as these conditions are associated with a high rate of mortality. The time of onset of the disease, delay of presentation in an emergency, and modalities of home remedies adopted before the presentation are key factors determining the outcomes of surgery. Other factors such as the age of the patient, general condition, adequate resuscitation, delay in surgery, comorbid conditions, complications due to anesthesia, and care after surgery all contribute toward the success of a surgery. In this study, the incidence of mortality and morbidity because of laparotomy was studied.

## Materials and methods

This study was conducted in a district hospital (secondary care institute), where only one surgeon was working. A total of 174 patients visited the hospital and participated in this study after providing informed consent. Patients presenting with complaints of acute abdomen and requiring laparotomy based on clinical judgment and investigations were included in the study. Patients whose general condition was not good due to septic shock, those with severe comorbidities (American Society of Anesthesiologists (ASA) class 3 and above), and those possibly requiring ventilatory support postoperatively on clinical and biochemical assessment were excluded and referred to a tertiary center. The study data were reviewed from April 2007 to January 2011 and March 2014 to February 2016 when the author was posted in the same government hospital. The data were entered in an MS Excel spreadsheet and analysis was done using MS Excel.

## Results

**Figure 1:**
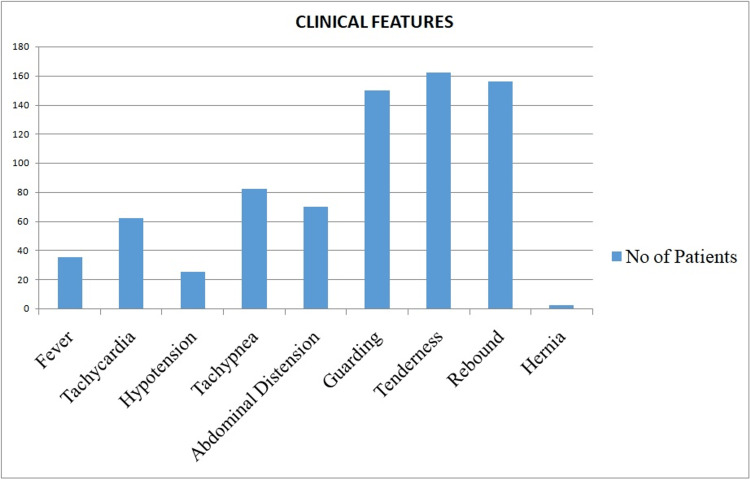
Clinical features of patients taken for surgery.

This study was conducted among 174 patients who presented in the emergency department with acute abdomen and were later subjected to emergency laparotomy in view of their clinical condition and laboratory and radiology investigations. On analysis, the majority of the patients, 46% (n = 80), were in the 21-40-year age group, followed by 32.75% (n = 57) in the 41-60-year age group, 14.94% (n = 26) in the less than 21-year age group, and 6.3% (n = 11) in the 61-80-year age group. Among the 174 cases, 76.43% (n = 133) were male and 23.56% (n = 41) were female patients. Comorbidity was present in 26.4% (n = 46) of patients mainly in the form of diabetes, hypertension, respiratory issues, etc. Figure 1 shows the clinical features of the patients who participated in the study.

In this study, the most common indication of laparotomy was perforation peritonitis, and the most common cause was duodenal perforation. Mortality was associated with the etiology of perforation peritonitis such as colonic perforation bearing greater microbiological load leading to early septicemia and chances of death. Another factor associated with mortality was the physiological age of patients. Most laparotomies were done within 24 hours of admission to the hospital.

Investigations

**Figure 2:**
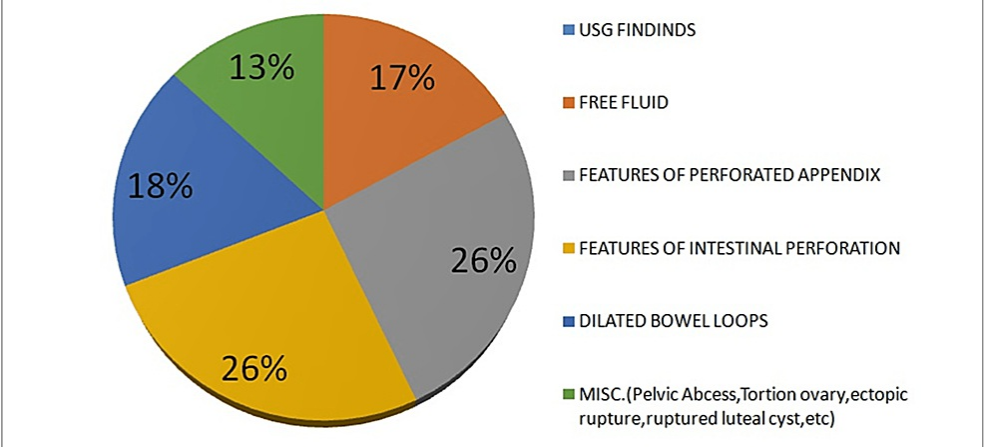
Ultrasonography findings of patients.

Among all patients, 32.75% (n = 57) had hemoglobin <10 g/dL, 41.4% (n = 72) had total leucocyte count >11,000 cells/mm^3^, and deranged renal functions were present in 22.9% (n = 40). X-rays of the chest and abdomen in standing and supine positions were taken in all cases, and 62.6% (n = 109) had free gas under the diaphragm. Many air-fluid levels were observed in 12.6% (n = 22), dilated gut loops were seen in 8.6% (n = 15), and no specific findings were noted in 16.1% (n = 28) of patients. Ultrasounds were done in 40.2% (n = 70) of patients. Ultrasound findings are shown in Figure 2.

Single-slice CT was done in 22.4% (n = 39) of patients, and the findings are tabulated in Table 1. Intestinal perforation which was not picked up by ski-gram was detected on a CT scan in 3.4% (n = 6) of patients. Once the diagnosis was established and the need for laparotomy was confirmed, surgery was done in 85.6% (n = 149) of patients within 24 hours and in 14.4% (n = 25) of patients after 24 hours. Patients were classified into 14 types of different diagnoses. These diagnoses are tabulated in Table 2 according to their frequency. The most common diagnosis was a duodenal perforation, which was present in 47.1% (n = 82), followed by gastric perforation in 13.8% (n = 24). Postoperatively, 65.5% (n = 114) of patients did not develop any complications and were comfortably discharged between days four and eight, depending upon the medical facility available at their remote village for minor wound care and removal of sutures. The most common complication encountered was wound sepsis. Distributions of postoperative complications are presented in Table 3.

**Table 1:** Contrast-enhanced CT abdomen findings.

CT abdomen findings	Frequency	Percentage
Not done	135	77.6
Features of perforation	06	3.4
Features of intestinal obstruction	11	6.3
Free fluid	12	6.9
Dilated gut loops	10	5.7

**Table 2:** Frequency distribution based on the postoperative diagnosis.

Postoperative diagnosis	Frequency	Percentage
Prepyloric/gastric perforation	24	13.79
Duodenal perforation	82	47.12
Ileal/jejunal perforation	09	5.17
Appendicular perforation	22	12.64
Small bowel obstruction	21	12.06
Intussusceptions	02	1.14
Obstructed hernia	01	0.57
Meckel’s diverticular pathology	04	2.29
Mesenteric ischemia	02	1.14
Appendicular abscess	02	1.14
Trocar-induced gut injury	01	0.57
Twisted ovarian cyst	01	0.57
Ruptured hemorrhagic cyst	01	0.57
Ruptured ectopic pregnancy	01	1.14

**Table 3:** Frequency distribution of postoperative complications.

Postoperative complications	Frequency	Percentage
Wound infection	27	15.51
Wound dehiscence	03	1.72
Wound sepsis with enterocutaneous fistula	03	1.72
Intra-abdominal abscess	01	0.57
Chest complications (pneumonia, acute respiratory distress syndrome)	17	9.77
Acute coronary syndrome	01	0.57
Keloids	02	1.14
Incisional hernia	03	1.72

In three patients, re-laparotomy was done between days three and four due to a leak of closure/anastomosis, and two patients died on days four and six of the second surgery due to sepsis. A total of three patients died during their hospital stay, all were above 65 years of age. Two died due to sepsis in the group which was operated on after 24 hours of presentation, and the third was due to sudden cardiac arrest. In patients with acute abdomen where free gas was identified on radiography in the peritoneal cavity, no further radiological investigations were done to make the decision for laparotomy. It was also observed that among 24 patients who were operated on after 24 hours of surgery, 14 developed postoperative complications, as shown in Table 4.

**Table 4:** Postoperative complications in patients operated on after 24 hours.

Complications	Frequency
Wound sepsis	7
Wound sepsis with fistula	2
Respiratory complications	3
Wound dehiscence	2

## Discussion

The main indication for laparotomy is either for trauma patients or acute abdomen. With the advent of modern diagnostic and precise investigations, the incidence of laparotomy, especially for trauma patients, has decreased. The decision to open the abdomen most often depends on the surgeon’s experience and clinical acumen aided with necessary investigations. These emergency laparotomies are also called exploratory laparotomies because most often the final diagnosis is only established by opening the abdomen. Our study is also different from other similar studies as it was conducted at a district hospital level, where only one surgeon was working without any other support from juniors or seniors. Undue delay to make the diagnosis on the pretext of exhaustive investigations is not justified; however, attention needs to be paid to resuscitating the patient on arrival at the hospital. The term acute abdomen encompasses a variety of disorders, which may be minor to life-threatening and require indoor admissions and sequential and coordinated workups [4]. It has been estimated that around 50% of admissions in surgical wards are for acute abdomen. Acute appendicitis, acute cholecystitis, peptic ulcer disease, acute pancreatitis, gastrointestinal perforations, small and large bowel obstruction, and gynecological disorders are among the common acute abdominal conditions [5]. Mortality can be caused by gastrointestinal perforation which requires surgical intervention. The size, site, and duration of perforation determine the severity of peritonitis. Physiological status, time since last meal, and coexisting diseases also determine the outcomes of surgery [6-8]. The type and burden of contamination depend on the anatomical location of intestinal perforation. From the proximal to the distal end of the alimentary canal, microbiological contamination increases. The acidic, pancreatic, and biliary secretions create a hostile environment in the stomach and duodenum which leads to a bacterial load of at least 103 organisms per gram of luminal content. In the jejunum, the content is 104 organisms per gram, and in the colon 1,012 per gram of luminal content [7,8]. The microorganism load is affected by the toxicity in the composition fluid in the organ. The gastric and intestinal secretions contain acidic contents or erosive pancreatic and biliary fluid, whereas the distal small bowel and colon contain relatively neutral contents [8]. This explains the early presentation of patients with gastric and duodenal perforation and the severity of pain due to chemical peritonitis compared to colonic perforations [9,10].

A detailed, synchronized history and clinical examination are crucial in identifying the anatomic source of pathology in gastrointestinal perforation. It also helps in determining the severity of the disease, evaluating the surgical risk, and guiding additional diagnostic testing [9,11,12]. Radiographic investigations of patients with acute abdomen include three views consisting of an upright chest and abdominal radiograph and a supine abdominal radiograph. The presence of free intraperitoneal gas signifies perforation. Studies have shown that even 1 mL of free intraperitoneal gas may be detected on a good-quality radiograph. However, plain radiographs have a sensitivity of 50-70% and the probable site of perforation cannot be defined [13]. Ultrasound is another modality used in groups where radiation hazards are to be taken into consideration such as pregnant women and children; however, it is not very definitive in considering free peritoneal air. CT has an upper edge over other modalities as multidetector CT can detect free gas as well as determine the site of perforation with an accuracy of 86% [10,14]. The definitive treatment for gastrointestinal perforation comprises fluid resuscitation, antibiotic treatment, control of the source, support of the organ system, and nutritional support [15,16]. Diagnostic delay or inappropriate management of intestinal obstruction becomes a surgical emergency that is associated with high mortality. Obstruction of the large intestine constitutes around 80% of all obstructions in the intestine.

Principal management for obstruction includes control of symptoms such as vomiting and pain by giving adequate analgesia, decompression and correction of dehydration, dyselectrolytemia by giving fluid and electrolytes, and surgery if required [17].

In our cohort, the majority of patients, 46% (n = 80), were in the 21-40-year age group, followed by 32.75% (n = 57) in the 41-60-year age group, 14.94% (n = 26) in the less than 21-year age group, and 6.3% (n = 11) in the 61-80-year age group. Similar to our study, in the studies by Chetri et al. and Laal et al., the younger age groups had a preponderance of acute abdomen and were subjected to exploratory laparotomy [18,19].

Among the etiologies leading to laparotomy, in our study, perforation peritonitis was the most common and observed in 66.08% of cases, including duodenal, pre-pyloric, and gastric and small intestinal perforation. However, appendicular perforation and abscess were observed in 13.78% of cases in which laparotomy was done. Small bowel obstruction due to bands and adhesions was observed in 12.06% of cases. Contrary to our study, other studies have reported acute appendicitis to be the leading cause of acute abdomen in 55% and 56.8% of cases who were subjected to laparotomy [18,19]. Similar to our study the literature shows visceral perforation and bowel obstruction in 50-60% and 15-20% of cases of laparotomy, respectively [20].

For more than two-thirds of the patients with small bowel obstruction with adhesions, conservative treatment is suggested, with initial attempts made to manage the patient conservatively. The first 48 hours are crucial in the conservative management of small bowel obstruction. Surgery is indicated in all patients in whom the following findings are observed: (1) the patient had not been settled despite conservative treatment, (2) the primary cause of obstruction such as hernia and obstructing carcinoma; (3) ominous signs of strangulation start to appear [17].

Limitations

This is a single hospital-based study, exclusively catering to nearly two districts, but more such studies are needed for the generalizability of results. Patients with acute abdomen who are toxic (ASA III category) and those with blunt trauma to the abdomen were not included and referred to tertiary centers because of inadequate resources, which prevented the consideration of the whole cohort of the acute abdomen in this study.

## Conclusions

The clinical experience of a surgeon and basic investigations are needed for the decision to proceed with laparotomy, instead of sophisticated time-consuming investigations which only increase the time lag for surgery which reciprocate in the form of added morbidity and outcomes of surgery.
